# Magnitude of under-nutrition among under five children in Ethiopia based on 2019 Mini-Ethiopia Demographic and Health Survey: Generalized Linear Mixed Model (GLMM)

**DOI:** 10.1186/s40795-022-00598-5

**Published:** 2022-10-17

**Authors:** Temam Beshir Raru, Galana Mamo Ayana, Bedasa Taye Merga, Belay Negash, Alemayehu Deressa, Abdi Birhanu, Fila Ahmed Hassen, Kedir Teji Roba

**Affiliations:** 1 School of Public Health, College of Health and Medical Sciences, Haramaya University, P.O. Box: 235, Harar, Ethiopia; 2 School of Medicine, College of Health and Medical Sciences, Haramaya University, Harar, Ethiopia; 3 School of Nursing and Midwifery, College of Health and Medical Sciences, Haramaya University, Harar, Ethiopia

**Keywords:** Malnutrition, Under-five, EDHS, GLMM, Ethiopia

## Abstract

**Introduction:**

Malnutrition is a major public health problem that is experienced by many developing countries, like Ethiopia. Though some studies were conducted to identify the magnitude and determinants of acute malnutrition among under-five children, there is a lack of evidence that is representative of all children in Ethiopia. Hence, this national-level data could be representative of all targets and provide us with updated information on the nation-wide magnitude of nutritional status among children under the age of five in Ethiopia.

**Methods:**

This study used data from the 2019 Mini-Ethiopia Demographic and Health Survey (EDHS). Children aged 0–59 months with anthropometry data were included. Data processing and analysis were performed using STATA 15 software. Cross-tabulations and summary statistics were done to describe the study population. Generalized Linear Mixed Models (GLMMs) were used to estimate the association between nutritional status and explanatory variables and were expressed as an odds ratio with a 95% confidence interval (CI). Model comparison was done based on Akaike and Bayesian information criteria (AIC and BIC).

**Results:**

The magnitude of stunting was 37.71% [95%CI: 36.35–39.08], while the magnitude of wasting was 7.14% [95%CI: 6.52–7.91]. Living in Tigray [AOR = 2.90, 95%CI: 2.05–4.11], Amhara [AOR = 1.98, 95%CI: 1.41–2.79], having a child aged 24–35 [AOR = 3.79, 95%CI: 3.07–4.68], and being a rural resident were all significantly associated with stunting. Being born in Tigray [AOR = 1.75, 95% CI: 1.02–3.01], being born into the richest family [AOR = 0.74, 95% CI: 0.27–0.80], and being born from mothers aged 25–29 [AOR = 0.73, 95% CI: 0.55–0.96] were all significantly associated with wasting.

**Conclusion:**

The magnitude of stunting and wasting is relatively high in Ethiopia. Region, place of residence, and age of the child were significantly associated with stunting, and region, wealth index, and age of the child were significantly associated with wasting. This result provides a clue to give due consideration to under-five children to mitigate the risks of malnutrition through various techniques.

## Introduction

Malnutrition is a major public health problem that is experienced by many developing countries. It stands as a consequence of several key social and economic factors such as lack of education, inadequate health care services and ill-informed cultural behaviors, acute lack of food, a recent attack of illness, inappropriate child care or feeding practices, or a mixture of these factors [1, 2]. Malnourished children have weakened immunity, are exposed to long-term developmental interruptions, and have a range of 5 to 20 times greater risk of death than well-nourished children [3]. The problem can be a primary cause of child death or a concomitant cause by profoundly increasing the mortality rate in children hit by common childhood diseases such as diarrhea, pneumonia, or measles. Children’s malnutrition may result in unfavorable long-term health, learning, and economic achievements [4].

Globally, it was assumed that 52 million under-five children were wasted, of which 17 million were harshly wasted. Mainly in the developing nations [5] and wasting affects 50 million children under the age of five, accounting for nearly half of all deaths in Asia and Sub-Saharan Africa [6–8]. Worldwide, one out of four children and one-third of children under five years of age in low and middle-income countries—and in Ethiopia, more than 2 out of every 5 children—are estimated to be stunted [9–11]. Stunting will have irreparable effects on a child’s future growth, which will increase population vulnerability and weaken its capacity to cope with episodes of food stress.

Children who are severely wasted are nine times more likely to die than their peers [6, 12–14]. The Ethiopian government launched an updated national nutrition program (NNP II) in 2016 to end hunger in Ethiopia by 2030 and has made various efforts to realize the Seqota declaration. One of the goals of the declarations is to overcome malnutrition among children by optimal breastfeeding and complementary feeding, prevention of micronutrient deficiencies, deworming, food fortification, and management of acute malnutrition [15]. The causes of child malnutrition are numerous and multidimensional; they vary from region to region. Breastfeeding initiation within one hour after delivery was considered the primary cause of malnutrition [16]. Furthermore, acute malnutrition in children is caused by complex interactions of various factors such as diarrhea or acute respiratory infection (ARI) and the absence of toilets for households [17], household food insecurity [18, 19], not washing hands after defecation/using toilet, hand washing before food preparation, dietary diversity, household wealth index, child immunization, number of under five children in the household and MUAC of the mother [19], prelacteal feeding [16, 20, 21], antenatal care services [16, 22–26], time of complementary feeding initiation & age of the child [16, 19, 20, 22, 27], maternal feeding during pregnancy [16], ever used family planning [20] and treating drinking water [28]. Though some studies in Ethiopia were conducted to identify the magnitude and determinants of acute malnutrition among under-five children, most of them used small sample sizes and covered small geographic areas. Hence, this national-level data could be representative of all targets and provide us with updated information on the nation-wide magnitude and determinants of nutritional status among children under the age of five in Ethiopia.

## Methods

### Source of data

This study used data from the 2019 Mini-Ethiopia Demographic and Health Survey (EDHS), which was conducted by the Central Statistical Agency (CSA) of Ethiopia, with technical support from ICF. The data was obtained from the measure demographic and health survey program (DHS) at www.measuredhs.com after justifying the reason for the request. The surveys employ a stratified, multistage, random sampling design. Detailed survey techniques and methods of sampling used to collect data have been recorded elsewhere [29].

This study included children aged 0–59 months with anthropometry data in the analysis of determinants of nutritional status among children under the age of 5 in Ethiopia. Children with no anthropometric data from stunting conditions or wasting conditions were also omitted from the analysis.

### Measurements

#### The outcome variables

Under-nutrition which has three commonly used indicators; stunting, wasting and underweight [30]. However, being underweight is the composite of stunting and wasting, so this study used stunting and wasting for the analysis.

#### Stunting

Children whose height-for-age z-score is below minus 2 (−2.0) standard deviations (SD) below the mean on the WHO Child Growth Standards [31].

#### Wasting

Children whose weight-for-height z-score is below minus 2 (−2.0) standard deviations (SD) below the mean on the WHO Child Growth Standards [31].

### Independent variables

#### Individual-level variables

are sex of a child, age of a child in months, birth order, breastfeeding status, mothers current age, mothers age at first birth, number of under-five children, mother’s educational status, number of household members, number of antenatal care visits, sex of household head, household wealth index, source of drinking water, time to get to a water source, type of cooking fuel, and type of latrine.

#### Water sources

Categorized into improved and unimproved according to WHO/UNICEF guidelines [32, 33].

#### Toilet facilities

were recoded as either improved (flush to piped sewer system, to septic tank, to pit latrine, ventilated improved pit latrine, pit latrine with slab, composting toilet) or non-improved (pit latrine without slab/open pit, bucket toilet, and hanging toilet/latrine) [32].

#### Household fuel types

were categorized as modern fuels (charcoal, electricity, natural gas, biogas, and kerosene) and traditional fuel (wood, animal dung, and other crops and straw) [32].

#### Community-level variables

are residence and region.

### Data processing and management

The extracted data were further cleaned, recoded, labeled. Furthermore, data were weighted so that the sample was representative of 0–59-months-old children in 2019 mini EDHS. STATA 15 software was used for data processing and analysis.

### Statistical analysis

Descriptive statistics were conducted using frequency and percentages for categorical measurements, and mean with standard deviation and median with inter-quartile range were used to summarize continuous variables. The important assumptions such as chi-square assumption, multicollinearity, and independence of errors were checked on top of the statistical modeling. Since the nature of data was hierarchical, therefore, two-level multilevel logistics regression was applied to identify the influence of individual-level and community (cluster) level variables. Four different models were fitted; Model-I was the multivariable model adjustment for individual-level variables, and Model-II was adjusted for community-level factors. In model-III, the outcome variable was equipped with potential candidate variables from both individual and community-level variables. The model with small Akaike Information Criteria (AIC) and Bayesian Information Criteria (BIC) was selected as the final model. The fixed effects (a measure of association) were used to estimate the association between nutritional status, i.e., stunting and wasting, and explanatory variables, and are expressed as an odds ratio with a 95% confidence interval. Regarding the measures of variation (random-effects), community variance with standard error and intra-cluster correlation coefficient (ICC) were used. Variables with a p-value less than 0.05 in the final model were statistically significant determinants of stunting and wasting.

## Results

### Descriptive characteristics of study participants

The study included a total of a weighted sample of 4,840 mothers with children aged 0 to 59 months. Among these, 50.90% of the children were male and 20.01% of them were less than 12 months old. Almost half of the children (49.5%) were ever breastfed but are not currently breastfeeding. Stunting was less common among children younger than 12 months compared to other age categories, whereas wasting was less common among children aged 36–47 months, compared to other age categories. The majority of the mothers were found in the age groups of 25–29. 63.81% of them gave their first birth at the age of less than twenty, 53.34% had no education, and 40.28% were from Oromia (Table 1).

**Table 1:** Characteristic of the study participants by stunting, and wasting and their overall sociodemographic status, Mini-EDHS 2019 (n = 4,840)

Variables	Weighted Frequency(%)	Stunting	Wasting
**Sex of child**			
Male	2464(50.90)	925(37.56%)	172(7.00%)
Female	2376(49.10)	900(37.85%)	173(7.30%)
**Age of child (months)**			
Less than 12 months 12–23 months 24–35 months 36–47 months 48–59 months	968(20.01) 931(19.24) 985(20.34) 1046(21.60) 910(18.81)	209(21.60%) 314(33.73%) 460(46.77%) 440(42.06%) 402(44.11%)	77(7.94%) 73(7.82%) 72 (7.28%) 49(4.72%) 75(8.22%)
**Birth order**			
First 2 to 4 5 or higher	1031(21.31) 2135(44.11) 1674(34.58)	411(39.87%) 818(38.34%) 595(35.56%)	65(6.32%) 159(7.44%) 122(7.26%)
**Breastfeeding**			
Ever breastfeed Never breastfeed Still breastfeeding	2,418(49.95) 241(4.99) 2,181(45.06)	867(35.85%) 115(47.65%) 843(38.65%)	185(7.63%) 15(6.43%) 146(6.67%)
**Number of under-five children**			
1 child 2 child 3 and above children	33(0.67) 1,965(40.61) 2,842(58.72)	9(28.45%) 695(35.36%) 1,121(39.44%)	3(9.63%) 107(5.46%) 235(8.28%)
**Mothers age (years)**			
15–24 25–29 30–34 35–39 40–49	1,116(23.05) 1,529(31.59) 1,065(22.01) 735(15.19) 395(8.16)	395(35.43%) 585(38.25%) 389(36.52%) 290(39.51%) 166(41.89%)	79(7.06%) 103(6.76%) 73(6.89%) 66(8.99%) 24(6.09%)
**Mothers educational status**			
No education Primary Secondary Higher	2,582(53.34) 1,733(35.80) 348(7.19) 177(3.66)	998(38.64%) 638(36.80%) 150(43.02%) 40(22.46%)	208(8.06%) 106(6.09%) 11(3.22%) 21(11.74%)
**Mothers age at 1st birth**			
Less than 20 20 and above	3,088(63.81) 1,752(36.19)	1,119(36.22%) 706(40.33%)	217(7.03%) 129(7.34%)
**Number of ANC visits (n = 3499)**			
No visit 1 to 3 visit 4 and above visit	871(24.89) 1,112(31.78) 1,516(43.33)	334(38.31%) 440(39.53%) 562(37.09%)	65(7.50%) 82(7.34%) 83(5.47%)
**Region**			
Tigray Amhara Oromia SNPPR Developing regions Addis Ababa, Diredawa, and Harari	348(7.19) 942(19.45) 1,949(40.28) 959(19.82) 469(9.69) 172(3.56)	171(49.06%) 399(42.42%) 668(34.28%) 385(40.18%) 161(34.32%) 40(23.22%)	32(9.20%) 79(8.40%) 84(4.32%) 56(6.02%) 88(18.68%) 5(2.90%)
**Residence**			
Urban Rural	1,224(25.29) 3,616(74.71)	392(31.98%) 1,433(39.64%)	77(6.31%) 269(7.43%)
**Sex of household head**			
Male Female	4,206(86.89) 634(13.11)	1,587(37.74%) 238(37.47%)	290(6.91%) 55(8.72%)
**Household wealth index**			
Poorest Poorer Middle Richer Richest	1,120(23.14) 1,065(22.01) 903(18.65) 848(17.52) 904(18.68)	415(37.06%) 430(40.33%) 326(36.08%) 344(40.54%) 311(34.40%)	128(11.39%) 66(6.20%) 43(4.73%) 66(7.78%) 43(4.80%)
**Source of drinking water**			
Improved Unimproved Others	3,016(62.32) 1,780(36.78) 44(0.91)	1,118(37.06%) 694(39.00%) 13(29.21%)	209(6.92%) 135(7.58%) 2(4.46%)
**Type of toilet**			
Improved Unimproved others	790(16.32) 3,992(82.48) 58(1.20)	309(39.19%) 1,492(37.38%) 23(39.74%)	49(6.26%) 292(7.31%) 5(7.94%)
**Type of cooking fuel**			
Modern Traditional Others	253(5.23) 4,560(4.21) 27(0.55)	94(37.06%) 1,720(37.72%) 11(41.92%)	11(4.44%) 333(7.30%) 2(6.74%)

### Prevalence of undernutrition

The prevalence of stunting was 37.71% [95%CI: 36.35–39.08]. Of these, 12.40% [95%CI: 11.51–13.36] were severely stunted. The prevalence of wasting was 7.14% [95%CI: 6.52–7.91], of which 1.40% [95%CI: 1.10–1.77] were severely wasted (Table 2).

**Table 2:** Prevalence of stunting and wasting among under 5 children in Ethiopia, 2019 (n = 4,840)

Types of Under-nutrition	Weighted frequency	Percentage(%)	Confidence Interval(95%)	
			**Lower**	**Upper**
**Stunting**				
No stunting	3,015	62.29	60.92	63.64
Stunting Moderate stunting Severe Stunting	1,825 1,225 600	37.71 25.30 12.40	36.35 24.10 11.51	39.08 26.55 13.36
**Wasting**				
No wasting	4,494	92.86	92.09	93.55
Wasting Moderate wasting Severe wasting	346 278 68	7.14 5.75 1.40	6.52 5.13 1.10	7.91 6.44 1.77

### Model selection

When we compared the three models for both stunting and wasting, Model-III, i.e., the model adjusted for both individual and community-level factors, had smaller AIC and BIC for both response variables. Hence, this model was the most parsimonious model, and the interpretation of the fixed effects for both stunting and wasting was based on Model-III (Table 3).

**Table 3:** Selection of most parsimonious model based on selection criteria

	Stunting		Wasting	
**Models**	**AIC**	**BIC**	**AIC**	**BIC**
**Null-Model**	6353.88	6366.88	2932.95	2945.96
**Model-I**	6162.23	6318.29	2909.76	3059.32
**Model-II**	6296.21	6348.23	2886.47	2938.49
**Model-III**	**6122.09**	**6317.17**	**2885.92**	**2890.49**

**NB: AIC =** Akaike Information Criteria and **BIC =** Bayesian Information Criteria

### Factors associated with stunting

The results from the final multivariable model (model adjusted for both individual and community-level factors) showed that region, place of residence, and age of the child were significantly associated with stunting. In the random effect, community variance = 0.17, SE 0.04, and 5.01% of the total variance of stunting can be ascribed to the community.

The odds of stunting were 2.90 and 1.98 times more likely among children whose mothers were from Tigray and Amhara, respectively, compared to those who were from Addis Ababa, Diradawa, and Harari [AOR = 2.90, 95%CI: 2.05–4.11] and [AOR = 1.98, 95%CI: 1.41–2.79]. The likelihood of stunting was 3.79 times more likely among children in the age group of 24–35 months compared to those who were less than 12 months [AOR = 3.79, 95%CI: 3.07–4.68] (Table 4).

### Factors associated with wasting

The results from the final multivariable model (model adjusted for both individual and community-level factors) showed that region, household wealth index, and age of the child were significantly associated with wasting. In the random effect, community variance = 0.27, SE 0.07, and 7.49% of the total variance of wasting can be ascribed to the community.

Our study shows that wasting was 75% more common among children whose mothers were from Tigray, compared to those who were from Addis Ababa, DireDawa, and Harari [AOR = 1.75, 95% CI: 1.02–3.01]. The risk of wasting was 0.47 times less likely for children who are from the richest families, compared to those who are from the poorest families [AOR = 0.77, 95% CI: 0.27–0.80] (Table 4).

**Table 4:** Factors associated with stunting, and wasting among fewer than 5 children in Ethiopia, 2019

Study Variables	Categories	Stunting	Wasting
		**AOR(95%CI)**	**AOR(95%CI)**
**Region**	Addis Ababa, Diredawa, and Harari Tigray Amhara Oromia SNPPR Developing regions	**1** 2.90(2.05–4.11) *** 1.98(1.41–2.79) *** 1.38(0.99–1.92) 1.58(1.13–2.21) ** 1.33(1.01–1.75) *	**1** 1.75(1.02–3.01) * 1.62(0.94–2.79) 0.68(0.38–1.23) 1.11(0.63–1.94) 2.15(1.40–3.29) ***
**Residence**	Urban Rural	**1** 1.37(1.05–1.81) *	**1** 0.75(0.49–1.13)
**Education**	No education Primary Secondary Higher	**1** 0.95(0.81–1.11) 0.78(0.59–1.03) 0.79(0.55–1.13)	**1** 0.76(0.58-1.00) 0.52(0.31–0.90) 1.34(0.77–2.33)
**Sex of child**	Male Female	**1** 0.93(0.82–1.05)	**1** 1.19(0.97–1.45)
**Household Wealth index**	Poorest Poorer Middle Richer Richest	**1** 1.01(0.83–1.23) 1.01(0.81–1.26) 1.09(0.86–1.39) 1.07(0.78–1.47)	**1** 0.66(0.47–0.92) * 0.60(0.41–0.88) * 0.70(0.47–1.02) 0.47(0.27–0.80) **
**Mothers age (years)**	15–24 25–29 30–34 35–39 40–49	**1** 0.95(0.80–1.13) 0.85(0.700–1.03) 0.95(0.76–1.19) 1.10(0.84–1.44)	**1** 0.73(0.55–0.96) * 0.77(0.57–1.05) 0.61(0.42–0.88) * 0.63(0.41–0.99) *
**Sex of HH head**	Male Female		**1** 1.00(0.77–1.30)
**Age of child (months)**	Less than 12 months 12–23 months 24–35 months 36–47 months 48–59 months	**1** 2.38(1.91–2.96) *** 3.79(3.07–4.68) *** 3.27(2.65–4.04) *** 2.97(2.40–3.67) ***	**1** 0.90(0.65–1.23) 0.79(0.57–1.08) 0.79(0.57–1.08) 0.82(0.60–1.12)
**Type of toilet facility**	Improved Unimproved Others	**1** 1.08(0.87–1.34) 0.69(0.21–2.22)	**1** 0.94(0.66–1.35) 1.00(0.12–7.81)
**Type of cooking materials**	Modern Traditional Others	**1** 0.87(0.67–1.22) 0.71(0.17-2.89)	**1** 1.22(0.65–2.30) 0.88(0.08–9.82)
**Source of drinking water**	Improved Unimproved Others	**1** 1.11(0.98–1.26) 0.84(0.43–1.61)	
		**Stunting**	**Wasting**
**Random effects** **Community Variance(Standard error)**		0.17(0.04)	0.27(0.07)
**ICC%**		5.01	7.49

NB: *** = Significant at P-value < 0.001, ** = Significant at P-value < 0.01, * = Significant at P-value < 0.05

ICC = Intra-class Correlation Coefficient, AOR = Adjusted Odds Ratio

## Discussion

This study investigated the magnitude and factors associated with under-nutrition among children under the age of 5 in Ethiopia based on national data. The prevalence of stunting was 37.71%, of which 12.40% were severely stunted and 7.14% had wasting. This finding is comparable to reports from Tanzania (28%) [34], Kenya (39%) [35], Vietnam (44% of stunting) [36], Chitwan (22.7% underweight, 37.3% stunting, and 25.7% wasting) [37], Belahara VDC (underweight, 27% stunting, 37%, and wasting, 11%) [38]. Children’s nutritional problems could be higher in low socioeconomic status population settings [39, 40].

In fact, low socioeconomic status can result in problems with nutritional status of children that could come from low intake of a balanced diet [41], irregular diet intake [42], low health care coverage. Despite the fact that Ethiopia has developed strategies such as the Growth and Transformation Plan (GTP), National Nutrition Plan (NNP), Seqota Declaration (SD), National Food Security Strategy, Nutrition Sensitive Agriculture (NSa) strategy, School Health and Nutrition Strategy (ShNS), Productive Safety Net Program (PSNP), and Food Safety and Quality related regulatory activities [43], these strategies are not functioning well.

The results of the multivariable logistic regression showed that region, residence, age of the child, and household wealth index were significantly associated with child nutritional status.

In this study, administrative region was found to be significantly associated with stunting. The odds of stunting were 2.90 and 1.98 times more likely among children whose mothers were from Tigray and Amhara regions, respectively, compared to those who were from Addis Ababa, Dire Dawa, and Harari regions. This result was consistent with other studies in Ethiopia [44–46] and other African Countries [47]. This could be due to the socio-economic status, residence, and feeding style variations across the urban-rural divide in different aspects [48]. This could also be policy implementation differences across the major cities; others could cause the differences. Moreover, this could be due to limited accessibility of hunger-fighting activities in areas far from cities, which could probably be due to lack of infrastructure and instabilities in some remote areas.

In this study, place of residence was also found to be significantly associated with stunting. This result is consistent with a study of cross-sectional data from the 2016 Ethiopian Demographic and Health Survey that showed children whose parents reside in rural areas had higher odds of childhood stunting than in urban areas [49]. This report is supported by the study from Northern part of Ethiopia [50], and Mozambique [51]. This could be due to the possible socioeconomic status variation across urban and rural areas, and the rural population may require additional resources to be treated in the case of seeking medical care. Malnutrition may result from a delay or refusal to seek medical care for whatever reason. In contrast, another report revealed that urban-residing children had a higher risk of getting malnourished than rural ones [52]. This may be due to better-equipped urban health-care systems, feeding practices, tradition or culture, sanitation conditions, and higher access to health-care facilities. Furthermore, the current study argued that there was no relationship between children’s nutritional status and their residence. [53, 54]. These contradicting ideas could be due to the fact that nutritional status is majorly affected by the living standards of the community regardless of where they live. The nutritional disparity across the two areas (urban and rural) could vary and cause different effects on the nutritional status of the children [55].

The study revealed that childhood stunting was found to be significantly associated with the age of the child; that stunting was 3.79 times more likely among children in the age group of 24–35 months compared to those who were less than 12 months. This revealed that as the child’s age increases, the risk of being stunted also increases. This finding is consistent with research from Ethiopia [56] and other African Countries [57]. The possible reason might be that caring practices usually tend to decrease when children are older than infants and are shifted to adult foods.

In terms of wasting, this study found that the household wealth index was significantly associated with wasting among children under the age of five. The risk of wasting was 0.47 times less likely for children who are from the richest families, compared to those who are from the poorest families. This result is consistent with different study results from different parts of Africa [53, 58], Peru [40], and Bangladesh [59]. This may result in children living in poorer conditions not getting enough food, not having a sanitary living environment, being more susceptible to infections, and not having access to basic health services due to the unaffordability of the services and other related costs.

Finally, this study reported that the age of a child was significantly associated with the wasting of under-five children. This result is consistent with different study results in African countries which indicate the prevalence of severe wasting is higher at younger ages and then declines and reaches a plateau by 24 months from previous studies in Ethiopia [60, 61]. This could be the variation of the age at which mothers discontinued feeding their children their breast milk, started complementary feeding and feeding styles, and the types of food the mothers fed their babies.

### Strengths and limitations of the study

The study used national data collected from nine regions and two city administrations, which enhanced its representativeness and this, can be considered strength of the study. Due to the cross-section design of the study, it is difficult to establish a temporal association between under-nutrition and other independent variables. Besides, recall bias can occur when reporting the age of the child.

## Conclusion

The magnitude of stunting and wasting are relatively high in Ethiopia which indicates that under-nutrition is public health concern among children. In addition, region, place of residence, and age of the child were significantly associated with stunting and region, household wealth index, and age of the child were significantly associated with wasting. Improving the socioeconomic status of households through economic empowerment would help to decrease the prevalence of malnutrition among children under age of five. Addressing the children through nutritional supplements and education in all regions and place of residences (both rural and urban) would contribute to reduction of under nutrition in Ethiopian children.

## Data Availability

The EDHS data sets are open and can be accessed from the Measure DHS website (www.measuredhs.com) through an online request by explaining the objective of the study.

## Declarations

The DHS research protocol complies with the Helsinki declaration.

## Abbreviations

AIC: Akaike Information Criteria.
BIC: Bayesian Information Criteria.
CI: Confidence Interval.
CSA: Central Statistical Agency.
DHS: Demographic and Health Survey.
EDHS: Ethiopia Demographic and Health Survey.
GLMM: Generalized Linear Mixed Model.
ICC: Intra-class Correlation Coefficient.
WHO: World Health Organization.
